# An emergency care research course for healthcare career preparation

**DOI:** 10.1186/s12909-021-02635-6

**Published:** 2021-04-12

**Authors:** Karen F. Miller, Rishub K. Das, Ciera D. Majors, Hadassah H. Paz, Ayana N. Robinson, Veronica F. Hamilton, Brittney E. Jackson, Sean P. Collins, Alan B. Storrow

**Affiliations:** 1 Vanderbilt University Medical Center, Nashville, USA; 2 Vanderbilt University School of Medicine, Nashville, USA; 3 University of Maryland Medical Center, Baltimore, USA; 4 Columbia University, New York, USA; 5 Wake Forest School of Medicine, Winston-Salem, USA

**Keywords:** Pre-medical, Undergraduate, Education, Shadowing, Immersion, Clinical, Research, Mentorship

## Abstract

**Background:**

University students have limited opportunities to gain healthcare clinical exposure within an academic curriculum. Furthermore, traditional pre-medical clinical experiences like shadowing lack active learning components. This may make it difficult for students to make an informed decision about pursuing biomedical professions. An academic university level research course with bedside experience provides students direct clinical participation in the healthcare setting.

**Methods:**

Described is a research immersion course for senior university students (3rd to 5th year) interested in healthcare and reported study enrollment with final course evaluations. The setting was an adult, academic, urban, level 1 trauma center emergency department (ED) within a tertiary-care, 1000-bed, medical center. Our course, “Immersion in Emergency Care Research”, was offered as a university senior level class delivered consecutively over 16-weeks for students interested in healthcare careers. Faculty and staff from the Department of Emergency Medicine provided a classroom lecture program and extensive bedside, hands-on clinical research experience. Students enrolled patients in a survey study requiring informed consent, interviews, data abstraction and data entry. Additionally, they were required to write and present a mock emergency care research proposal inspired by their clinical experience. The course evaluations from students’ ordinal rankings and blinded text responses report possible career impact.

**Results:**

Thirty-two students, completed the 16-week, 6–9 h per week, course from August to December in 1 of 4 years (2016 to 2019). Collectively, students enrolled 759 ED patients in the 4 survey studies and reported increased confidence in the clinical research process as each week progressed. Ranked evaluations were extremely positive, with many students describing how the course significantly impacted their career pathways and addressed an unmet need in biomedical education. Six students continued the research experience from the course through independent study using the survey data to develop 3 manuscripts for submission to peer-reviewed journals.

**Conclusions:**

A bedside emergency care research course for students with pre-healthcare career aspirations can successfully provide early exposure to patients and emergency care, allow direct experience with clinical bedside research, research data collection, and may impact biomedical science career choices.

**Supplementary Information:**

The online version contains supplementary material available at 10.1186/s12909-021-02635-6.

## Background

Pre-health undergraduate and graduate students often make important career choices early and seek out clinical experiences to make more informed decisions. Such exposure allows students to gain experience interacting with patients, conduct research, and understand the nuances of their healthcare career of choice. Unfortunately, early clinical exposure is often unavailable to students, largely due to increasing requirements surrounding the protection of patient confidentiality [1]. Further, there is a lack of immersive clinical experiences allowing students to take on an active role in a clinical setting. More common are passive, observational learning experiences, often termed “shadowing”. While these programs provide value, having an active role in patient interactions and clinical research has the potential to provide students with greater appreciation for different healthcare roles and their impact on patient experiences. The dearth of immersive exposure has been recognized not only by undergraduate institutions nationwide, but also by medical schools, who have begun to develop new curricula to provide clinical experiences to medical students as early as the first year [2].

When immersive clinical experiences are available to pre-health students, they have been reported to be formative in assessing career fit. For instance, a one-credit course where premedical students conducted patient interviews, used problem-solving skills to report potential diagnoses, and analyzed retrospective data sets has been described [3]. The Minneapolis Heart Institute Foundation Summer Research Internship Program provides cardiology shadowing and clinical research experiences to students, culminating in scholarly contributions to peer-reviewed journals and presentations at major research conferences [4]. An experiential learning program has provided internships for students as nursing aides, clinical assistants, and health educators [5]. An undergraduate educational program for students working as ED research assistants has been described in terms of providing a departmental framework for patient enrollment [6]. These programs have provided students with a better understanding of their intended healthcare career.

An adult emergency department (ED) is an excellent environment for direct clinical experiences, as it can provide students with exposure to ambulatory patients, a wide variety of clinical problems, and broad perspectives on numerous societal issues [1, 6]. Such experiential learning requires students to actively and iteratively apply and reflect on the knowledge, concepts, and skills required for emergency care and clinical research. It provides opportunities to confront the uncertainties, complexities, and challenges of caring for the acutely ill and injured and apply such experiences to career choices.

We describe curriculum development and conduct of a course addressing the fundamentals of clinical care and providing senior-level university students interested in a healthcare career with an active, career-informing clinical research experience.

## Methods

Our course was an 16-week, 6–9 h per week, course offered from August to December, in 1 of 4 years (2016 to 2019), to senior-level university students interested in healthcare professions. The clinical setting was an adult, academic, urban, level 1 trauma center Emergency Department (ED) within a tertiary-care, 1000-bed Medical Center. The course provided students exposure to emergency medicine care through a classroom lecture program (Table 1) and an extensive bedside, hands-on clinical research experience.

**Table 1:** Schedule of didactics and other course-related activities for one semester

Didactics Schedule Fall Immersion Course Course Orientation, Practical Informed Consent in the Emergency Care Setting	
Introduction to US Emergency Care; COBRA, EMTALA, and Caring for the Underinsured	
Past Student Reflections; Student Emergency Care Research Project	
Emergency Operations Study Group Fluid Resuscitation in Adults: Research Design, Methodology, and Results	
Geriatric Emergency Care Research: Performing Clinical Research in the Elderly	
Clinical Research Proposal: Questions, Aims, and Hypotheses	
Clinical Research Proposal Workshop (students present)	
Emergency Care Operations Research, Center Overview	
Clinical Trauma Research	
Clinical Toxicology Research	
Who, What, When, Where of Air Transport	
Research Evaluating Patients for Cardiovascular Emergencies	
Clinical Research in Pediatric Emergency Care Optional: attend emergency medicine journal club for faculty and residents	
Individual Research Mentoring Meetings	
Clinical Research Proposal: formal presentations	

*COBRA* Consolidated Omnibus Budget Reconciliation Act, *EMTALA* Emergency Medical Treatment & Labor Act

The course objectives were to enable students to: 1) describe the emergency care system in the United States, including provisions for disadvantaged populations and the uninsured; 2) understand the historical development of human subject research protections; 3) describe clinical research data collection methods, storage, and basic analytic techniques. 4) understand the role clinical research plays filling in critical knowledge gaps in patient care; and 5) apply these concepts to the bedside through identification and enrollment of patients in ongoing clinical studies or trials. An interactive, academic, web-based learning environment (Brightspace, D2L Corporation), was utilized to manage course material and assignments.

Prior to starting the course, students were required to complete training in human subject research conduct, healthcare information privacy and security, and clinical emergency care safety. Further prerequisites included security and confidentiality agreements to obtain a hospital picture ID badge, immunizations and tuberculin testing as required by employee health, and access to the electronic health records.

Outside of 100 min of weekly lectures, students were required to perform 12, 4-h, weekly ED shifts, with the emergency care research team, as academic research assistants. With initial supervision, students were responsible for conducting a local Institutional Review Board (IRB) approved, survey research study consenting ED patient or caregiver volunteers. The students were scheduled in pairs and were provided supervision by a designated clinical research preceptor during their clinical shift. Successful completion of a clinical informed consent checklist (Supplementary Material) was required prior to the student being able to independently approach ED patients or their caregivers for research. Studies for the 4 years included a life status interview on patient perspectives of their current health and alternative medicine therapies, methods of personal medication reconciliation, a survey to determine patient and caregiver perspectives of wait times and ED flow, and patient practices surrounding hypertension engagement and medication adherence. Students conducted all aspects of subject identification, research patient or surrogate consent, data management, database configuration, data entry and data quality assessment.

The ten weekly, two-hour, classroom lectures focused on emergency care delivery, human subject research, clinical research design, data collection and introductions to specific faculty research interests (Table 1). Sessions were presented by emergency medicine faculty and experienced staff specializing in emergency care research. In lieu of a final exam, students developed an emergency care research study with a two-page written and mock oral research proposal presented to the faculty and class during one of the last class sessions.

**Fig. 1:**
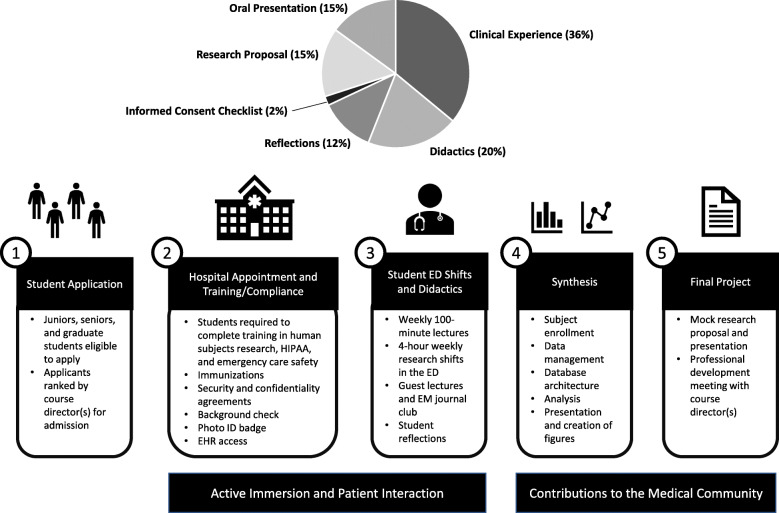
Grading rubric for the Immersion Course (top). Students are evaluated based on the primary course objectives with an emphasis on active participation and presence in clinical shifts at the ED. The ability to obtained informed consent and demonstrate appropriate bedside manner is captured in the “Informed Consent Checklist.” The program model for the Immersion Course is summarized (bottom). (1) Juniors, seniors, and graduate students affiliated with the Medicine, Health, and Society department at Vanderbilt were invited to apply and were evaluated by the course director(s). (2) Accepted students underwent an on-boarding process to receive an appointment at the hospital and electronic health record (EHR) access. (3) Students work 4-h weekly shifts in the ED and engage in didactic seminars. (4) The data collected from students and the ED research team is organized and analyzed before (5) students complete and present a final project

A 100-point grading scale rubric (Fig. 1) was established based on clinical research participation (36%), class participation (20%), clinical shift experience reflections (12%), an informed consent checklist (2%), and completion of the emergency care research proposal in written (15%) and oral (15%) formats. A final course evaluation consisted of both individual quantitative measurements and qualitative written evaluations by students of the instructors and course, based upon standard local university methodology.

Emergency Medicine clinical investigators provided a survey study for each cohort of 6–10 students per course offered each year. A different investigator and subject survey study was provided for each student cohort. During the extensive bedside experience, the students had a clinical preceptor available each shift.

This report was determined exempt from review by the local, university Institutional Review Board. The student data collection utilized the secure web application for building and managing the online surveys, Research Electronic Data Capture (REDCap) [7].

## Results

### Student demographics

Course participants consisted of 32 students enrolled from course inception in 2016 through 2019, a total of four Fall session cohorts with 6–10 students per cohort The students were selected senior-level, 3rd-5th year, of their 4 or 6 year programs in the Center for Medicine, Health and Science (MHS) university program. The selection was based on desire to take the course, academic standing and Fall schedule recommendations from program professors through the application process. Of the total 32 students, 25 (78%) identified as female and 7 identified as male (22%). Most students that completed the course were in 4-year programs, 24 (75%).

### Course evaluation

**Fig. 2:**
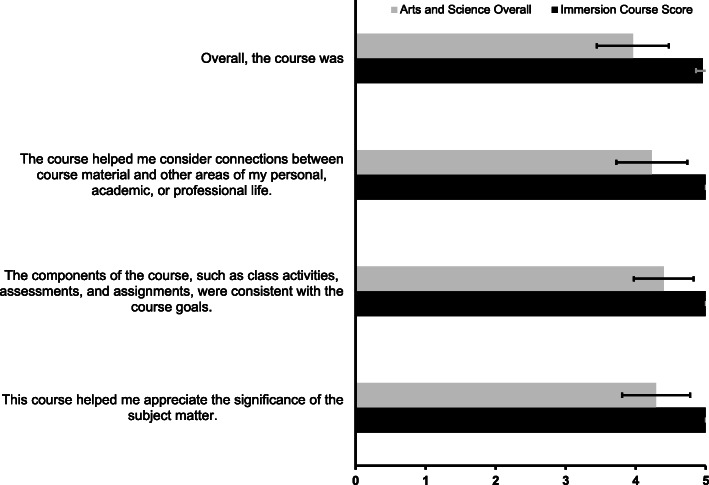
Agreement and sentiment toward course evaluation statements from 4 years of the Immersion Course compared with the 2019 aggregate scores from all coursed in the College of Arts and Science. A score of 5 corresponded to “Strongly Agree” or “Excellent” and a score of 1 corresponded to “Strongly Disagree” or “Poor”

Of 32 students, 26 completed a course evaluation survey (81%). To determine the success, utility, and reception mean scores and standard deviation for course-related survey questions were presented in comparison to mean scores for all courses in the Vanderbilt School of Arts and Science in 2019 (Fig. 2). The ordinal rankings from 1 through 5 are a metric for student satisfaction; 1 indicates “Poor” or “Strongly Disagree” and 5 indicates “Excellent” or “Strongly Agree.” Unanimously, the students felt that the curriculum and course activities were aligned with the course objectives (5.00/5.00 vs 4.40/5.00) and helped foster a greater appreciation for clinical medicine and research (5.00/5.00 vs 4.29/5.00). Furthermore, all students indicated that the course encouraged introspection and offered meaningful connections to a professional career in medicine or personal aspirations (5.00/5 vs 4.23/5.00). An overwhelming majority of students rated the course as “Excellent” overall (4.96/5.00 vs 3.96/5.00). For all study questions, the Immersion Course received superior responses to the aggregate responses from Vanderbilt Arts and Science courses in 2019.

### Student narratives

Students were also invited to provide written feedback throughout in clinical shift experience reflections and evaluations at completion of the course. To determine how the course affected student attitudes toward a career in biomedicine, 3 course instructors reported responses to anonymous, open-ended survey questions at course completion as well as assigned reflective questions during the course.

Students most addressed the clinical, “hands-on” features of the course and described their experiences working in the ED as “impactful,” “unique,” and “immersive.” Many students indicated that the immersion component of the course helped reaffirm their professional interests in medicine or healthcare. One student reported,

*“This course was truly one of the most impactful and profound experiences I’ve had at Vanderbilt, and it has fundamentally shaped the way I view medicine, research, and patient care. [The Instructor] went above and beyond to provide us with an opportunity that I don’t think could be found anywhere else, and I am confident that the skills and knowledge I learned will help guide me in my future career.”*

The opportunity to play an active role in patient interaction was contrasted with previous experiences in other courses, organizations, and shadowing. Students commented the course offered a breadth and magnitude of clinical exposure that is “hard to gain as an undergraduate,” especially within the confines of a structured curriculum.

*“There is no other class or organization on campus that will give pre-med or pre-nursing students this type of practical experience. Through the clinical shifts each week, I have reaffirmed my desire to pursue medical school.”*

For a few, the course not only provided perspective on a career in medicine but also imparted learnings about conducting research and the role of research in patient care. One student indicated that she originally “didn’t want to pursue research after medical school.” But after being “immersed in the world of clinical research,” she has “developed a new goal for [her] future as a physician.” Another student commented,

*“The active learning immersion through this class has been nothing short of life changing. I feel like this course was helpful for a career in medicine, but also for developing my understanding of research as a whole.”*

Nearly all students characterized the guest lectures as favorable and different from typical curriculums because each lecturer was “very specialized in their field and passionate about what they do which was very encouraging to a future healthcare provider.”

Most students also cited the impact of interactions with nurse and physician mentors throughout the course as “invaluable” and “incredibly encouraging.” Generally, students felt supported by medical center faculty and felt like “Vanderbilt should have more classes like this one that ties undergraduates to the medical center.” Several students requested letters of recommendation from the course professor for admission to biomedical graduate programs, most commonly medical school.

## Discussion

We describe an emergency care research course for students with pre-healthcare career aspirations providing exposure to patients and clinical research. A unique aspect of our course compared to shadowing was students serving as research team members during clinical shifts. A staff member of the employed research team also served as clinical research preceptor and provided hands-on training, safety, and security. The preceptor assured good research study conduct, yet, gave the students an immersive experience of being a research team member accountable for their group research study project versus the observation role in shadowing.

Using student satisfaction as a metric, results from course evaluations corroborate the pragmatism associated with implementing an immersive clinical research course. The students who enrolled in the course were actively engaged clinical research and care, interacting with patients and a variety of healthcare professionals including emergency department physicians, nurses, and research associates. The immersive clinical environment encourages a deeper appreciation for academic medicine and impact career trajectories.

Furthermore, some students are affected by social determinants that influence their availability for extracurricular activities outside of an academic course such as shadowing or research [8]. For those students, a course designed to offer structured immersion in clinical settings may be uniquely valuable and enhance applications to graduate school programs, addressing inequities in medical education.

Some limitations are noted. This course was originally conducted without intention for research publication; course evaluations were distributed according to our institution’s established protocol. Such evaluations are utilized for all university courses and were not designed to address novel aspects of a beside clinical experience. Student feedback and responses were voluntary, although our participation rate was over 80%. Minor changes in course didactics and participating faculty occurred each year and student feedback were used to modify the clinical experience both within years and between years.

## Conclusions

Our experience and data may drive new collaborative programs between undergraduate and medical school educators, attracting more pre-health students to academic medicine.

An academic, senior-level university student clinical immersion course can readily provide early patient exposure to emergency care and research, including all aspects of human subject enrollment. Such experiences were evaluated favorably and described to address an unmet need in pre-health education.

## Supplementary Information

**Additional file 1.**

## Data Availability

The datasets used during the current study are not publicly available due the identifiability of the individual source but are available from the corresponding author on reasonable request.
