# Supplementary figures and images for: An emergency care research course for healthcare career preparation
Source: BMC Med Educ. 2021 Apr 12;21:206. doi: 10.1186/s12909-021-02635-6 (PMC8042954; doi:10.1186/s12909-021-02635-6)

Supplement


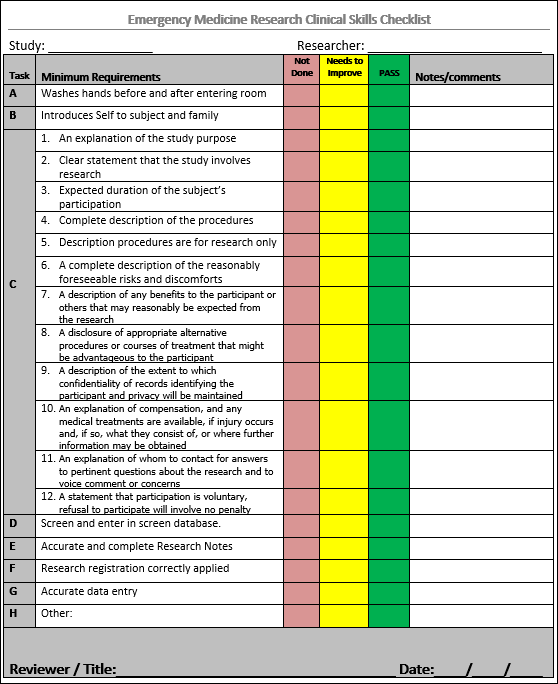

Supplement: Supplementary file 1 — Additional file 1. [file 12909_2021_2635_MOESM1_ESM.docx]
